# Preventing myopia in East Asia

**Published:** 2019-05-13

**Authors:** Jason James Ha, Mingguang He

**Affiliations:** 1 Medical Doctor: Centre for Eye Research Australia, Melbourne, Australia.; 2 Professor of Ophthalmic Epidemiology, University of Melbourne, Melbourne, Australia.

**Spending enough time outdoors can drastically reduce the risk that a child will develop myopia.**

**Figure:**
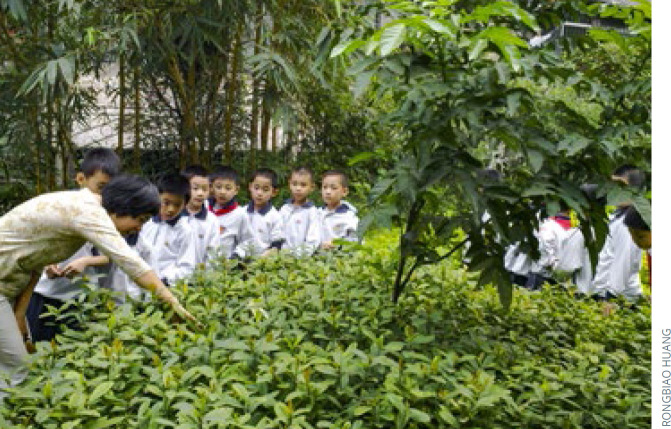
Outdoor activities help to delay the onset of myopia. CHINA

The prevalence of myopia among school-aged children has been increasing over several decades, reaching up to 80% among junior high school graduates in East Asian populations,^1^ particularly in China's most developed cities, Hong Kong, Taiwan and in those of Chinese descent living in Singapore.

To cope with this epidemic, risk factors and effective methods of prevention must be identified. Although increasing educational pressures and near work time have been highlighted as important risk factors for the increasing prevalence of myopia,^2^ it is unlikely that there will be a cultural shift in the focus on academic performance in these communities. Therefore, there is an urgent need to identify other modifiable risk factors for myopia.

Increased time spent outdoors was first proposed as a protective factor in a 3-year follow-up study^3^ and then reported in various studies, such as the Sydney Myopia Study,^4^ Orinda Study^5^ and a cohort study from Singapore.^6^ However, such reports can only prove association rather than a causal effect.

In 2009, a 3-year randomised trial in Guangzhou, China proved that an additional 40 minutes of outdoor activity at school reduced the 3-year cumulative incidence of myopia from 39.5% to 30.4%, a relative reduction of nearly 25%, among grade 1 primary school students.^7^ Another clinical trial in Taiwan suggested that 80 minutes of outdoor time per day could reduce the incidence by 50%.^8^ It is possible that outdoor intervention may have a dose-response preventative effect for myopia, though further studies are needed to substantiate this.^8^

A few approaches to maximise outdoor exposure have been suggested, given the fact that it is unlikely that class time will be reduced in the East Asian setting. One approach is to incorporate class time in an outdoor environment, while another is to lock classroom doors during break or play time (recess) which could add an additional 60 minutes of outdoor time.

**Figure 1:**
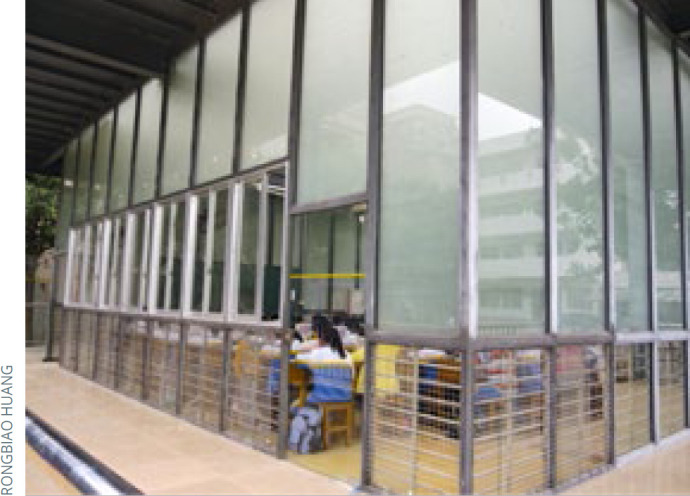
A bright classroom designed to delay the onset of myopia

Another approach is to increase in-classroom illumination during lessons. Our research group has proposed an innovative classroom design that incorporates a glass roof and walls in an attempt to maximise light intensity while students study indoors (Figure 1).^9^ We have also attempted to develop an LED lamp that resembles outdoor light intensity in the classroom. The efficacy of these two interventions are currently being investigated.

When school leaders or parents try to implement interventions that increase students' time spent outdoors, they must also consider protecting the students against sunburn or damage caused by ultraviolet radiation. A recent study in Taiwan suggests that a much lower illumination of 1,000–3,000 lux (equivalent to illumination levels under tree shade) is sufficient to generate a protective effect.^10^

Other interventions, such as targeting smart device screen time or eye exercises, are yet to be supported by scientific evidence.

China has recently proposed a country-wide myopia control strategy engaging both the education and health sectors, which involves government policy reform, involvement of schools and parents in myopia prevention, improved health services targeting myopia, and health promotion discouraging risky behaviours leading to myopia.^11^

The impact of increasing outdoor time is far-reaching. A 25% reduction in incidence among primary school students would mean a significant delay in the onset of myopia, and this could reduce the prevalence of myopia and perhaps high myopia in the wider population. The challenge is how to translate these findings into an intervention that can be delivered during day-to-day school activities.
